# High expression of Endogenous Retroviruses from intrauterine life to adulthood in two mouse models of Autism Spectrum Disorders

**DOI:** 10.1038/s41598-017-19035-w

**Published:** 2018-01-12

**Authors:** Chiara Cipriani, Laura Ricceri, Claudia Matteucci, Alessia De Felice, Anna Maria Tartaglione, Ayele Argaw-Denboba, Francesca Pica, Sandro Grelli, Gemma Calamandrei, Paola Sinibaldi Vallebona, Emanuela Balestrieri

**Affiliations:** 1 Department of Experimental Medicine and Surgery, University of Rome Tor Vergata, Via Montpellier 1, Rome, 00133 Italy; 2 Centre for Behavioural Sciences and Mental Health, Istituto Superiore di Sanità, Rome, Italy; 3 Institute of Translational Pharmacology, National Research Council, Via Fosso del Cavaliere 100, 00133 Rome, Italy

## Abstract

Retroelements, such as Human Endogenous Retroviruses (HERVs), have been implicated in many complex diseases, including neurological and neuropsychiatric disorders. Previously, we demonstrated a distinctive expression profile of specific HERV families in peripheral blood mononuclear cells from Autistic Spectrum Disorders (ASD) patients, suggesting their involvement in ASD. Here we used two distinct ASD mouse models: inbred BTBR T+tf/J mice and CD-1 outbred mice prenatally exposed to valproic acid. Whole embryos, blood and brain samples from the offspring were collected at different ages and the expression of several ERV families (ETnI, ETnII-α, ETnII-β, ETnII-γ, MusD and IAP), proinflammatory cytokines (IL-1β, IL-6 and TNF-α) and Toll-like receptors (TLR3 and TLR4) was assessed. In the two distinct mouse models analysed, the transcriptional activity of the ERV families was significant higher in comparison with corresponding controls, in whole embryos, blood and brain samples. Also the expression levels of the proinflammatory cytokines and TLRs were significantly higher than controls. Current results are in agreement with our previous findings in ASD children, supporting the hypothesis that ERVs may serve as biomarkers of atypical brain development. Moreover, the changes in ERVs and proinflammatory cytokines expression could be related with the autistic-like traits acquisition in the two mouse models.

## Introduction

Endogenous Retroviruses (ERVs), the major subset of retrotransposons, are the relics of ancestral retroviral infection to germline cells, stably integrated into the host cellular DNA, which comprise about 8% of the genome in humans and over 10% in mice^1,2^. Even if the transposition of retroelements is deemed responsible for the evolution and the genomic instability^3^, the vast majority of human ERVs (HERVs) sequences are biochemically inert and silenced by host cellular machineries. Their activity is tightly regulated during the life cycle of each individual, and the active propagation and random insertion into genomic DNA leads to gene alterations, with consequent uncontrolled expression and possible involvement in various diseases including cancer^4^, autoimmune^5^ and neurological and psychiatric disorders^6–8^. HERVs contribution to human diseases has been associated also to the interplay with immune system^9^. Among the neuro-developmental disorders, we observed altered expression of selected HERV families in children with Attention Deficit Hyperactivity Disorder (ADHD) and Autism Spectrum Disorders (ASD)^10–12^. The complex aetiology of ASD is still largely unknown, as both genetic susceptibility and environmental factors are involved, with epigenetic changes possibly playing a major role during early brain development^13,14^. It has also been described that immunological alterations seem to be involved in the pathophysiology of ASD^15,16^, since several ASD risk genes encode components of the immune-system^17^. Furthermore, a possible relationship between maternal immune activation and defects in fetal development was proposed, although a causal link has not been proved yet^18^.

As in humans, in the mouse species many families of ERVs have been identified including the most active intracisternal A-particle elements (IAPs)^19,20^ and Type II early transposons (ETns)^21^. IAPs are able to transpose through retroviral mechanisms in the cisternae of the endoplasmic reticulum^22^ and can influence the transcriptional profile of nearby genes, providing functional promoter elements and modulating local epigenetic landscape through changes in DNA methylation and histone modifications^23^. ETn elements are non-coding retroviral-like sequences, classified into ETnI and ETnII subtypes^24^. ETnII subtype carries long terminal repeats (LTR) and comprises mouse type D retrovirus (MusD), containing gag-pro-pol genes but lacking the env gene^25^, and ETnII-α, ETnII-β e ETnII-γ^26^. IAPs, and ETnII elements are responsible for about 10% of mouse spontaneous mutations by inserting into genes^21^. With the aim to support our previous observation that autistic behaviour in humans was associated with a distinctive transcriptional profile of some HERV families, detectable in peripheral blood mononuclear cells (PBMC) from ASD patients^10,11^, we assessed in whole embryos and, from the offspring at different time after birth, in blood and brain samples the IAPs and ETns expression in two distinct mouse models of ASD: BTBR T+tf/J inbred mice (referred as BTBR here-in) and CD-1 outbred mice treated *in utero* with the anticonvulsant and histone deacetylase inhibitor valproic acid (VPA). BTBR is an idiopathic model of ASD, displaying several behavioural traits relevant to ASD, such as impairments in social and communication domains, reduced cognitive flexibility and high levels of repetitive behaviours^27–29^ in comparison to the inbred C57BL6/J strain, considered as standard control strain for BTBR. CD-1 outbred mice, treated *in utero* with VPA, show behavioural ASD-like alterations including early motor hyperactivity^30^, social deficits and cognitive impairments^31,32^. Of note the use of VPA as antiepileptic medication during pregnancy, is associated with a significantly increased risk of somatic anomalies, ASD and other developmental disabilities in the offspring^31,33,34^. Multiple mechanisms are called upon to explain the therapeutic effects of VPA as well as its developmental neurotoxicity: direct interference with GABAergic neurotransmission, interaction with neural remodelling and neurogenesis, modulation of folate metabolism and free radicals’ production^35–37^. In rodent models, through epigenetic mechanisms prenatal VPA alters expression of several genes and proteins implicated in brain and immune system development^38,39^.

Finally, we also evaluated the transcriptional activity of proinflammatory cytokines (IL-1β, IL-6, TNF-α) and Toll-like receptors (TLR3 and 4), main actors of innate immunity, in whole embryos and, from the offspring at different time after birth, in blood and brain samples to investigate on the hypothesised link between the ERVs transcriptional activity and the immune system.

## Results

### High levels of ERVs expression in BTBR mice from intrauterine life till adulthood

**Figure 1:**
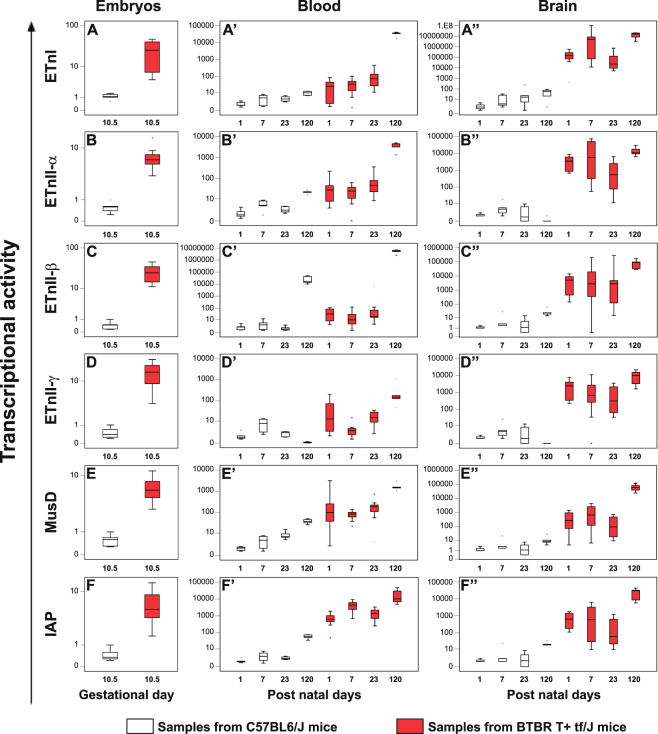
Transcriptional activity of ETnI, ETnII-α, ETnII-β, ETnII-γ, IAP and MusD genes in whole embryos and, from the offspring, in blood and brain samples obtained from BTBR and C57BL6/J mice. The ERVs expression profile in whole embryos (panel A–F) at gestational days 10.5, in blood (panel A’–F’) and brain (panel A”–F”) samples at post natal day 1, 7, 23 and 120 from BTBR (red box plots) and C57BL6/J (white box plots) mice, was examined by real time-PCR; depicting mild (black dot) and extreme (asterisk) outliers for each group were showed. In BTBR embryos and in blood and in brain samples, the expression levels of all ERVs, were significantly higher with respect to C57BL6/J mice.

The transcriptional activity of ETnI, ETnII-α, ETnII-β, ETnII-γ, MusD and IAP genes were analysed in whole embryos explanted at gestational day (GD) 10.5 and in blood and brain samples obtained at post natal day (PND) 1, 7, 23 and 120 from BTBR and C57BL6/J mice, by real time-PCR. The data obtained are represented as box plots in Fig. 1.

The expression levels of all ERV genes were higher (p ≤ 0.002) in BTBR (panels A–F, red box plots) than in C57BL6/J embryos (white box plots) and were maintained also after the birth, at all the age points examined, in blood (panels A’–F’) as well as in brain tissue (panels A”–F”). Particularly, the transcriptional activity of all ERV genes analysed was significantly higher at all age points in blood samples from BTBR mice (red box plots) than in age matched C57BL6/J mice (white box plots) (*p* ≤ 0.038), with the exception of ETnII-γ gene at PND 7 (panel D’). In C57BL6/J mice, except for ETnII-γ (which showed maximal expression at PND 7) the highest expression levels of genes analysed were found at PND 120 (*p* ≤ 0.008, PND 1, 7 and 23 *vs* PND 120). In BTBR mice the same age-dependent profile was evident (*p* ≤ 0.050, PND 1, 7 and 23 *vs* PND 120), but levels of expression attained at PND 120 were strikingly higher.

In brain samples from BTBR mice (red box plots), the transcriptional activity of all ERVs analysed was significantly higher than C57BL6/J mice at all age points (white box plots) (*p* ≤ 0.028). In C57BL6/J mice the highest levels of expression of ETnI, ETnII-β, MusD and IAP were observed at PND 120 (PND 1 *vs* PND 120 *p* ≤ 0.028), while those of ETnII-α and ETnII-γ at PND 7 (PND 1 *vs* PND 7 *p* ≤ 0.050). BTBR similarly reached the highest levels at PND 120, statistically different in the comparison with those found at PND 1, PND 7 and PND 23 (*p* ≤ 0.028), except for ETnII-γ (PND 1 *vs* PND 120) and ETnI, ETnII-α (PND 7 *vs* PND 120). See Supplementary Table S1 for median values of ERVs expression and Table S3 for *p*-values for all the possible comparisons.

### High levels of cytokines and Toll-like receptors were found in embryos and in blood and brain from BTBR mice

**Figure 2:**
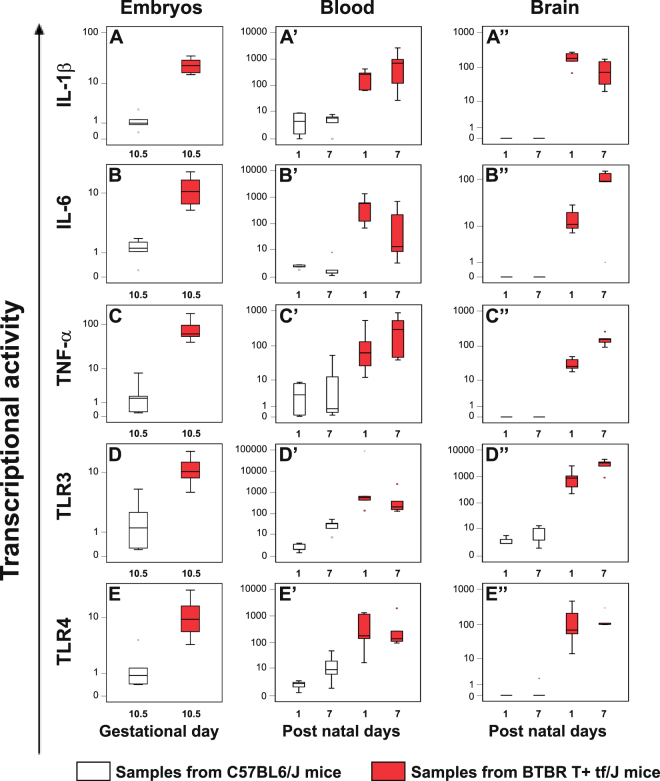
Expression levels of IL-1β, IL-6, TNF-α, TLR3 and TLR4 genes in whole embryos and, from the offspring, in blood and brain samples obtained from BTBR and C57BL6/J mice. The cytokines and TLRs expression in whole embryos (panel A–E) at gestational days 10.5, in blood (panel A’–E’) and brain samples (panel A”–E”) at post natal day 1 and 7 from BTBR (red box plots) and C57BL6/J (white box plots) mice, was examined by real time-PCR; depicting mild (black dot) and extreme (asterisk) outliers for each group were showed. In the BTBR embryos and in blood and brain samples, higher levels of cytokines and Toll-like receptors expression than in age-matched C57BL/J controls were detected.

In the same samples we evaluated the expression levels of IL-1β, IL-6, TNF-α, TLR3 and TLR4 genes, by real time-PCR, and the data obtained are represented as box plots in Fig. 2. Whole embryos explanted at GD 10.5 and blood and brain samples at PND 1 and PND 7 were taken into account.

The expression levels of cytokines and TLRs were higher in whole embryos from BTBR mice (panel A–E, red box plots) than in C57BL/J control groups (white box plots) (p ≤ 0.003) and the high levels observed were maintained after the birth both in blood (panel A’–E’, red box plots) (*p* ≤ 0.032) and in brain samples (panel A”–E”, red box plots) (*p* ≤ 0.028) (see Supplementary Table S2 for median values of cytokines and TLRs expression levels and Table S3). Interestingly, only in BTBR embryos, positive correlations between the expression levels of ERVs, cytokines and TLRs were found (see Supplementary Table S4). Particularly, positive correlation was revealed between the expression of IL-1β and ETnII-β and MusD (p ≤ 0.047); IL-6 and MusD (p = 0.021); TNF-α and ETnII-β, ETnII-γ, and MusD (p ≤ 0.047); TLR3 and ETnI (p = 0.047).

### Prenatal VPA exposure induced the expression of most ERVs analysed in embryos and in blood and brain samples from CD-1 mice

**Figure 3:**
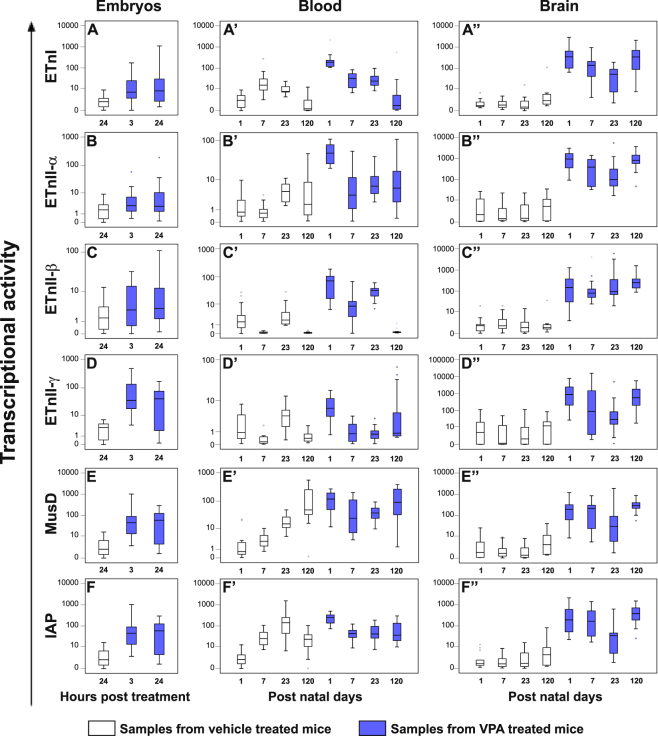
Transcriptional activity of ETnI, ETnII-α, ETnII-β, ETnII-γ, IAP and MusD genes in whole embryos and, from the offspring, in blood and brain samples obtained from VPA- and vehicle-treated CD-1 mice. The ERVs expression profile in whole embryos (panel A–F) explanted 3 and 24 hours post the treatment, in blood (panel A’–F’) and brain (panel A”–F”) samples obtained at post natal day 1, 7, 23 and 120, was examined by real time-PCR and represented as box plots, depicting mild (black dot) and extreme (asterisk) outliers for each group. In whole embryos higher transcriptional activity of all ERVs analysed in VPA groups (blue box plots) than in vehicle groups (white box plots) was evident, at both time points. In blood, VPA-treated mice (blue box plots) showed higher transcriptional levels of all ERVs than in age-matched controls (white box plots), at post natal day 1 and 7, while in brain at each age points.

The transcriptional activity of the same ERV genes mentioned above were analysed in whole embryos explanted 3 and 24 hours following VPA treatment at GD 10.5 and in blood and brain samples obtained at PND 1, 7, 23 and 120, by real time-PCR. The data obtained are represented as box plots in Fig. 3.

In whole embryos (panel A–F) higher transcriptional activity of most ERVs analysed at both time points after VPA treatment (blue box plots), was evident in comparison with corresponding values of whole embryos obtained from vehicle-treated dams (white box plots) (p ≤ 0.042), except for ETnII-β (panel C) and ETnII-α (panel B) genes at 3 hours and 24 hours post treatment, respectively (Tables S1 and S3).

The Spearman analysis revealed in vehicle embryos the presence of positive correlations between all ERVs expression levels (p ≤ 0.046); conversely, several of the correlations were lost after VPA treatment. However, both at 3 and 24 hours after VPA treatment, the positive correlations among the expression levels of two clusters of ERVs, including ETnI, ETnII-α and ETnII-β (p ≤ 0.046) and ETnII-γ, MusD and IAP (p < 0.001), were maintained (see Table S5).

After birth, the results showed that in the blood tissue (panel A’–F’), prenatal exposure to VPA, increased significantly the transcriptional activity of ERV families (blue box plots) at PND 1 and PND 7 (except for ETnI at PND 7, panel A’), compared to age-matched control groups (white box plots) (*p* ≤ 0.006). Moreover, in the VPA mice the highest levels of all ERVs were observed at PND 1 while a remarkable reduction of transcriptional activity was observed at PND 7 (*p* ≤ 0.050).

In brain samples (panel A”–F”) from VPA-treated mice (blue box plots), the transcriptional activity was instead always higher than age-matched control groups (white box plots) (*p* ≤ 0.010), in which ERVs show very low levels of expression that remain stable across ages (Tables S1 and S3).

### Prenatal exposure to VPA modified cytokines and Toll-like receptors expression already 3 hours after treatment as well as after the birth

**Figure 4:**
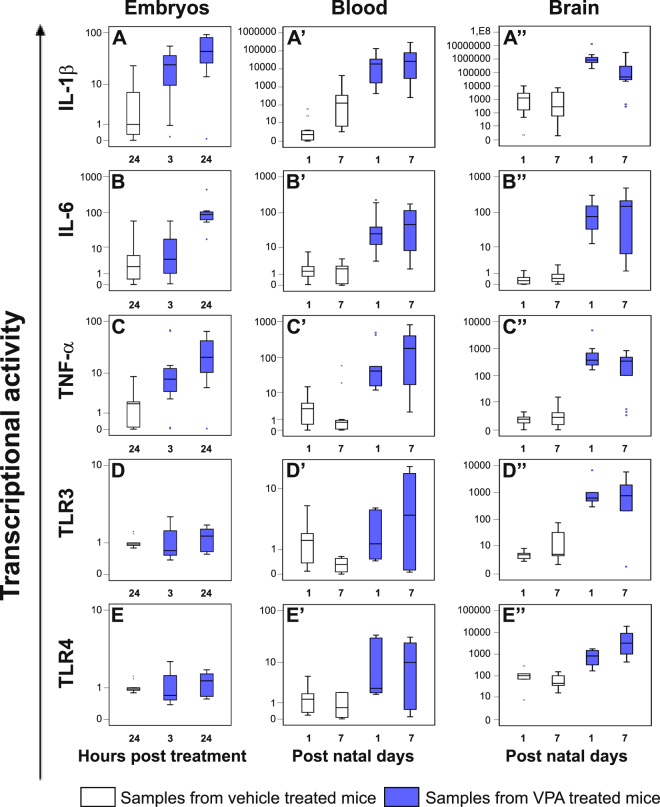
Expression levels of IL-1β, IL-6, TNF-α, TLR3 and TLR4 genes in whole embryos and, from the offspring, in blood and brain samples from VPA- and vehicle- treated CD-1 dams. The cytokines and TLRs expression in whole embryos (panel A–E) explanted 3 and 24 hours post the treatment, in blood (panel A’–E’) and brain (panel A”–E”) samples obtained at post natal day 1 and 7 from VPA- and vehicle- treated CD-1 dams, was evaluated by real time-PCR and represented as box plots, depicting mild (black dot) and extreme (asterisk) outliers for each group. Prenatal exposure to VPA at gestational day 10.5 increased cytokines expression in embryos (blue box plots), already from 3 hours post treatment than in controls (white box plots). In blood and in brain, also the expression levels of TLRs were higher in VPA mice (blue box plots) than in controls (white box plots).

In the same samples in which ERVs transcriptional activity was analysed, we evaluated the expression levels of IL-1β, IL-6, TNF-α, TLR3 and TLR4 genes, by real time-PCR. The data are represented as box plots in Fig. 4. Whole embryos explanted 3 and 24 hours following VPA injection at GD 10.5 and blood and brain samples at PND 1 and PND 7 were taken into account.

In embryos 3 hours post treatment with VPA (panel A–E, blue box plots), we observed a significant increase of IL-1β expression (p ≤ 0.005); after 24 hours, also IL-6 and TNF-α expression levels were higher compared to those observed in the embryos from vehicle-treated CD-1 dams (white box plots) (p ≤ 0.042) (see Tables S2 and S3). The correlation analysis highlighted a positive correlation between expression of IL-6 and EtnII-γ (p = 0.029) in embryos 3 hours post treatment with VPA (see Table S6).

In blood samples (panels A’–E’) the highest levels of expression of the cytokines and the TLR4 were found in the VPA-treated groups (blue box plots), at all ages considered (*p* ≤ 0.041).

In brain (panels A”–E”), levels of cytokines and Toll-like receptors transcriptional activity, were also higher in VPA-treated groups (blue box plots) than in age-matched control groups (white box plots) (*p* ≤ 0.005) and positive correlations between the expression of IL-1β and most of the ERVs, were evidenced by Spearman test in each treatment group (VPA group: ETnII-α, ETnII-γ, MusD and IAP, *p* ≤ 0.037; control group: ETnI, ETnII-α, ETnII-γ, MusD and IAP, *p* ≤ 0.020) (see Table S6).

## Discussion

Here we show for the first time in two distinct models of ASD [BTBR T+tf/J (BTBR) inbred mice and CD-1 outbred mice treated *in utero* with VPA] that the transcriptional activity of six different ERV families (ETnI, ETnII-α, ETnII-β; ETnII-γ, MusD and IAP) is significantly higher than in corresponding control mice (C57BL/J and CD-1 untreated mice, respectively), i.e. in whole embryos at about half of gestation (GD 10.5) and in blood and brain tissues at different postnatal ages.

Current results are in agreement with our previous data showing a distinctive expression profile of some human HERV families in blood samples from two different cohorts of young autistic patients^10,12^ and support the hypothesis that ERVs could be implicated in ASD.

In parallel, here we also report higher expression levels of IL-1β, IL-6 and TNF-α proinflammatory cytokines and TLR3 and TLR4, in the same whole embryos and in blood and brain samples from BTBR mice and VPA-treated mice. All together these data suggest that high levels of ERVs expression and inflammatory transcriptional profile are related, or at least proceed in parallel, with the autistic-like traits in the two mouse models.

Interestingly, the ERVs transcriptional activity was higher in BTBR than in C57BL6/J whole embryos and these high expression levels were maintained after the birth; in blood and in brain tissue of BTBR mice, ERVs transcriptional activity was higher in comparison to age-matched C57BL6/J mice at all considered ages, suggesting a long lasting activation of ERVs that could have an effect on brain functions throughout the life span.

Although the BTBR strain is a consolidated model for autistic-like behavioural signs, it has the weakness of being an inbred strain, thus lacking a univocal specific control strain, essential to compare transcriptional profiles. To circumvent this limitation we extended our analysis to another extensively validated mouse model of ASD, consisting in CD-1 outbred mice *in utero* exposed to the valproic acid (VPA), an established anti-convulsant drug whose use during pregnancy has been robustly associated with an increased risk of developing ASD in children^33,34^. VPA is a histone deacetylase inhibitor able to modulate HERVs expression, depending on cell type^40^. Notably, in embryos the treatment induced the increase of ERVs expression suggesting a rapid and direct effect of the VPA injection. In the vehicle group, positive correlations between all ERVs expression levels were found, supporting the physiological activity of the ERVs during embryogenesis as already described^41^. Worthy of note, after VPA treatment, several correlations were lost suggesting that in VPA-induced mouse model of ASD, the acquisition of the autistic-like traits could be linked to the dysregulation of ERVs activity, occurring during intrauterine life.

At variance from BTBR, the transcriptional activity of all ERVs was significantly higher in blood of VPA-treated animals compared to untreated controls only at PND 1, and the subsequent decrease may be attributed to the rapid turnover of blood cells. Conversely, in brain samples from VPA-treated mice the transcriptional activity of ERVs was significantly higher than in controls at each age point, and reached the highest values at PND 120. Indeed, the brain is a stable tissue characterized by slow/absent cellular turnover, and data obtained suggest that here VPA induces a permanent increase of ERVs expression, with a profile comparable to what observed in BTBR mice. The different time course of ERVs expression in the two different district in VPA treated offspring suggests that PBMCs can be reliable biomarkers also for brain atypical development at early life stages, provided metabolic specificities of the peripheral and central nervous system (CNS) district are considered. Moreover, ERVs transcriptional activity levels were substantially comparable in the two different control groups (C57BL6/J and CD-1) in embryos (p = 0.112) as well as in blood (p ≥ 0.196) and in brain samples (p ≥ 0.571). These observations strongly suggest the view that abnormally increased ERVs expression is associated with derailed brain development and with the acquisition of the autistic-like phenotype in the offspring. ERVs are usually repressed by two main mechanisms, DNA methylation occurring mostly in germ cells^42–44^ or various histone modifications occurring mostly in zygotes^45–47^. During the implantation stage these transient histone marks are again replaced by DNA methylation. Thus, histone modifications are usually responsible for the temporary repression of ERVs in transient stem cell populations, whereas DNA methylation is responsible for the more stable and permanent repression in further committed and differentiated cell populations^48–50^, such as brain cells. These findings also support the usefulness of multiple ASD mouse models, particularly to i) analyse in greater detail the prenatal phase, that is certainly crucial according to several recent ASD risk factor analysis^51^, ii) obtain selective information concerning the brain district, mostly inaccessible in human studies and, at the same time iii) to evaluate potential translational value of peripheral biomarkers.

Several robust etiopathological hypotheses for ASD include epigenetic events occurring in the prenatal period, when somatic and germ cells undergo a global remodelling that regulates cell differentiation and tissues specification^52,53^. Recently, in mice, it was found that the ERVs transcription regulation and expression silencing in neural progenitor cells is controlled in the first days of embryogenesis by TRIM28 (Tripartite motif-containing protein 28); only when TRIM28 is deleted the expression of ERVs is up-regulated^46,54^; further studies are needed to clarify the role of TRIM28 in ERVs upregulation observed in these two ASD mouse models, and to assess potential TRIM28 alterations in neurodevelopment.

The present study also reports increased expression *in utero* as well as in early postnatal development of some cytokines and Toll-like receptors in both ASD models. Epidemiologic studies provide evidence that maternal exposure to infections, stress or medications, greatly increases the risk for ASD in offspring^55,56^, by altering the immune status of the fetal brain and the fetal immune system^57^. Rodent models have definitively played an important role in establishing the maternal immune activation (MIA) as a causal factor, mediated by cytokines, for ASD-like symptoms in offspring^58–61^.

Actually, our results point to an immune dysregulation characterized by high levels of proinflammatory cytokines, such as IL-1β, IL-6 and TNF-α, already from intrauterine life in both models. The immune modifications were then maintained early after birth in both blood and brain samples from BTBR and VPA-treated mice, in agreement with human observations from autistic individuals^62–64^. Of note, in BTBR embryos several positive correlations were found between the expression of some ERV genes and the proinflammatory cytokines analysed, suggesting that ERVs activation and immune deregulation proceed in parallel.

As for BTBR, our results are consistent with the recent characterizations of this inbred mouse strain, revealing an inflammatory and immune profile higher than that of C57BL6/J control strain^65,66^, similarly to what observed in ASD children. In particular, brain levels of IL-6 transcriptional activity are very high in this mouse strain; interestingly, IL-6 appears to be a mediator of MIA effects upon brain development, because of its inhibitory effect on DNA methylation, possibly resulting in permanent epigenetic alterations of genes^67^.

As for VPA mice, HDACs inhibitory effects and epigenetic mechanisms of VPA could underlie its long-lasting consequences^35^. A rapid transient increase in histone acetylation has been described in the mouse embryonic brain, already two and four hours after VPA exposure at embryonic day 12.5, back to normal levels 12 hours after^30^. In this model, IL-1β transcriptional levels are high, both in blood and brain samples, in line with the role described for this cytokine in the MIA to produce ASD-like phenotype in the offspring^67^. Of note, the similarity between the two ASD models in developmental cytokine expression suggest that toxic mechanisms associated to oxidative stress may be implicated in their atypical neurobehavioural development. This would be in agreement with the known role of VPA on oxidative metabolisms^68^ that could run in parallel or trigger epigenetic modifications. Such hypothesis is worth to be investigated in future *in vivo* studies assessing protective interventions.

Finally, it has been widely described that during pregnancy maternal exposure to pathogens and unspecified damage signals induce innate immunity through interaction with TLRs, particularly with TLR3 and TLR4. Transcriptional activity of these TLRs in whole embryos from BTBR mice and in blood and brain from the both models is higher than in corresponding controls. This is in line with reports in schizophrenia, bipolar disorder and autism^69–71^.

The positive correlations between the IL-1β expression and most of the ERVs, in brain samples from both controls and VPA-treated mice, suggest an association between the ERVs and this single proinflammatory cytokine, but further studies are needed to deeply evaluate causal links between them.

Putative pathogenic effects mediated by ERVs in neurological and psychiatric conditions in humans have been already described^72^. ERVs may alter cellular function in the developing brain by means of multiple mechanisms, including modulation of DNA stability and transcription, alteration of cell signalling pathways, and activation of immune system^8^. Definitive proof that ERVs are the cause - and not an epiphenomenon- of neurodevelopmental disorders is still lacking, although data here reported support their involvement in ASD, and indicate a promising research avenue for novel diagnostic markers and even, potential early therapeutic approaches for ASD in the future.

## Methods

### Animals

Mice were either bred in Istituto Superiore di Sanità (ISS), Rome, Italy (BTBR and C57BL6/J, original breeders from Jackson, Maine and Charles River France, respectively) or purchased (CD-1 dams and sires, Harlan, S. Pietro al Natisone, Italy). Mice were housed in polycarbonate breeding cages with a 12hr light-dark cycle (light on 8:00 pm-8:00 am) and with free access to food and water. The VPA mice were produced by treating outbred pregnant CD-1 female mice with a single dose of VPA (500 mg/kg, sc) at gestational day (GD) 10.5. Time and dose of administration were selected among those not inducing maternal toxicity or frank malformations at birth but causing behavioural alterations resembling the ASD phenotype (^73^ for a comprehensive review). For these experiments, control mice were CD-1 mice whose pregnant mothers had been injected with saline at GD 10.5. VPA solution was prepared by dissolving VPA in 0.9% saline to a final concentration of 150 mg/ml.

The animals were analysed from intrauterine life to adulthood at different age points. To this purpose, 5 BTBR and 3 C57BL6/J dams were sacrificed at GD 10.5 to obtained 10 and 8 whole embryos, respectively. Blood and brain samples (at least 6 samples, from 3 or more different litters at each age points) were obtained from mice at postnatal day (PND) 1, 7, 23 and 120.

VPA- and vehicle-dams were sacrificed 3 and 24 hours after injection and whole embryos were collected: 26 samples from 3 different VPA-treated dams 3 hours after treatment and 20 VPA- and 16 vehicle-embryos from 3 dams 24 hours after treatment. Blood and brain samples from VPA- and vehicle treated offspring (at least 8 samples, from 3 or more different litters at each age points) were obtained from mice at PND 1, 7, 23 and 120.

### Ethical statement

This study was carried out in accordance with the Italian Animal Welfare legislation (D.L. 26/2014) that implemented the European Committee Council Directive (2010/63/EU); the experimental protocol 223-B/2011 was approved by Italian Ministry of Health.

### Tissue collection

The animals were euthanized and whole embryos were explanted. Blood samples were collected in heparinized tubes and stored at −80 °C and brains were removed from the skull, immediately frozen in dry ice and stored at −80 °C until use.

### RNA extraction from whole embryos and blood and brain samples

Total RNA isolation from whole blood samples was performed using NucleoSpin RNA Blood kit (Machenery-Nagel, Dueren, Germany) according to the manufacturer’s instructions and starting from 200 µl. When the volume of the sample was less than 200 µl it was added with phosphate-buffered saline. RNA isolation from whole embryos and brain samples was performed using NucleoSpinTriPrep (Machenery-Nagel) according to the manufacturer’s instructions and starting from 30 mg or less of tissue. Contaminating DNA was removed by a DNase treatment and all RNA samples were stored at −80 °C until analysis was performed.

### RT- Real time PCR

DNase-treated RNA obtained from whole embryos, blood and brain samples was reverse-transcribed into cDNA using Improm-II Reverse Transcription System (Promega, Fitchburg, Wisconsin, USA) according to the manufacturer’s protocol. For the reaction, 250 ng of RNA obtained from whole embryos and brain samples and an amount of RNA corresponding to 5 µl of initial blood sample, were used. The transcriptional levels of six ERV families (ETnI, ETnII-α, ETnII-β, ETnII-γ, MusD and IAP), three proinflammatory cytokines (IL-1β, IL-6 and TNF-α) and two Toll-like receptors (TLR3 and TLR4) were quantitatively assessed by Real-time PCR. The assays were performed in a Bio-rad instrument (CFX96 Real-Time System, Biorad, Hercules, California, USA) using SYBR Green chemistry (iTaq Universal SYBR green Supermix, Biorad) with specific primer pairs^26,74,75^. To set-up the real time reaction a serial dilution (10-fold) was done to calculate efficiencies and correlation coefficient, by formula [efficiency = 10 (^−1/slope^)] and all primer pairs used showed an efficiency ranging 0.96 to 0.98.

Real time PCR reaction included 0.20 µl of cDNA, forward and reverse primers at 150 nM each for ERVs and at 300 nM each for proinflammatory cytokines and for TLRs, 10 µl of iTaq Universal SYBR green Supermix, in a total volume of 20 µl. The reaction was conducted for 1 cycle at 95 °C for 3 minutes, then for 40 cycles at 95 °C for 45 seconds and at 60 °C for 1 minute. Each sample was analysed in triplicate and a negative control (no template reaction) was included in each experiment, to check out any possible contamination. The housekeeping glyceraldehyde 3-phosphate dehydrogenase gene (GAPDH) was used to normalize the results^26^. Each experiment was completed with a melting curve analysis to confirm the specificity of amplification and the lack of any non-specific product and primer dimer. Quantification was performed using the threshold cycle (Ct) comparative method: the relative expression was calculated as follows: 2^−[∆Ct (sample)−∆Ct (calibrator)^ = 2^−∆∆Ct^, where ∆Ct (sample) = [Ct (target gene) − Ct (housekeeping gene)]. In BTBR mice, for the analysis in whole embryos, the ∆Ct (calibrator) was the mean of ∆Ct of all embryos from C57BL6/J at GD10.5 while for the analysis of the blood and brain samples, the ∆Ct (calibrator) was the mean of ∆Ct of all brain or blood samples from C57BL6/J at PND1.

In VPA mouse model, for the analysis in whole embryos, the ∆Ct (calibrator) was the mean of ∆Ct of all vehicle-treated embryos obtained 24 hours after the treatment, while for the analysis of the blood and brain samples was the mean of ∆Ct of all brain or blood samples from vehicle-treated CD-1 mice at PND 1.

Real time PCR results were represented by box plots, depicting mild (black dot) and extreme (asterisk) outliers for each group.

### Statistical analysis

The Mann Whitney *U* test was used to compare the ERVs’, proinflammatory cytokines’ and TLRs’ transcriptional levels, analysed by quantitative real-time PCR, in whole embryos, blood and brain samples obtained from BTBR strain and related controls (C57BL6/J), offspring of VPA-treated (VPA group) and vehicle-treated dams (control group), respectively. To determine any correlation of different parameters, the Spearman’s rho correlation coefficient was calculated. Statistical analyses were carried out using SPSS software (version 20.0). Statistical significant comparisons were considered when *p* < 0.050.

### Data availability statement

All data generated or analysed during this study are included in this published article.

## Electronic supplementary material

Supplementary information
