# Supplementary material for: High expression of Endogenous Retroviruses from intrauterine life to adulthood in two mouse models of Autism Spectrum Disorders
Source: Sci Rep. 2018 Jan 12;8:629. doi: 10.1038/s41598-017-19035-w (PMC5766538; doi:10.1038/s41598-017-19035-w)
Supplement: Supplementary file 1 — Supplementary information [file 41598_2017_19035_MOESM1_ESM.pdf]

Supplementary information

High expression of Endogenous Retroviruses from intrauterine life to adulthood in two mouse models of Autism Spectrum Disorders.

Chiara Cipriani, Laura Ricceri, Claudia Matteucci, Alessia De Felice, Anna Maria Tartaglione, Ayele Argaw-Denboba, Francesca Pica, Sandro Grelli, Gemma Calamandrei, Paola Sinibaldi Vallebona, Emanuela Balestrieri.

Supplementary table S1. Median value and interquartile range (IQR) of the ERVs transcritional activity in C57BL6/J, BTBR, vehicle- and VPA-treated mice

			ETnI	ETnII-α	ETnII-β	ETnII-γ	MusD	IAP
**Whole embryos**								
	**GD 10.5**	C57BL6/J	1.11 (0.84-1.45)*	0.59 (0.32-0.81)	0.46 (0.17-0.75)	0.42 (0.24-0.89)	0.58 (0.29-0.84)	0.35 (0.25-0.79)
	**GD 10.5**	BTBR	26.09 (6.60-42.14)	6.46 (5.39-8.49)	22.95 (14.37-26.20)	14.50 (8.06-19.68)	6.10 (4.23-8.93)	5.24 (3.51-9.11)
**Blood**								
	**PND 1**	C57BL6/J	1.01 (0.75-2.24)	0.92 (0.49-2.50)	1.73 (0.57-3.06)	0.89 (0.61-2.12)	1.20 (0.64-1.50)	0.93 (0.76-1.67)
		BTBR	27.24 (2.96-70.46)	27.70 (6.66-64.91)	31.71 (6.03-95.97)	12.97 (2.61-118.85)	101.46 (34.87-330.42)	626.35 (372.58-1099.51)
	**PND 7**	C57BL6/J	4.40 (0.80-7.23)	4.44 (2.50-8.06)	3.06 (0.58-8.61)	7.40 (1.98-12.93)	4.14 (0.86-7.03)	3.03 (0.93-6.21)
		BTBR	34.28 (12.85-53.48)	24.48 (7.03-46.68)	24.12 (5.76-52.26)	2.91 (1.32-3.99)	86.37 (59.98-111.37)	4363.34 (1808.96-6931.73)
	**PND 23**	C57BL6/J	3.88 (2.42-5.59)	2.21 (1.54-3.76)	0.87 (0.53-1.78)	2.11 (0.96-2.44)	6.51 (5.70-10.67)	1.94 (1.65-2.79)
		BTBR	73.42 (24.84-150.36)	46.29 (19.50-79.75)	19.78 (16.01-80.58)	14.93 (7.60-28.93)	191.28 (98.82-244.63)	1352.37 (622.19-2232.53)
	**PND 120**	C57BL6/J	9.72 (6.53-12.35)	21.50 (18.64-24.89)	16809.63 (11917.83-46174.40)	0.07 (0.02-0.14)	37.58 (30.06-49.21)	59.47 (43.12-75.67)
		BTBR	34540.47 (25851.42-39126.47)	4317.56 (2222.23-4568.37)	5261344.18 (3764067.70-6637975.59)	150.17 (86.52-630.84)	1554.25 (1269.00-2299.96)	10219.24 (5825.05-38254.90)
**Brain**								
	**PND 1**	C57BL6/J	1.24 (0.56-3.36)	0.82 (0.72-1.81)	0.85 (0.69-1.84)	0.84 (0.69-1.84)	0.78 (0.68-2.08)	0.88 (0.72-1.66)
		BTBR	141040.75 (54479.72-331983.19)	3439. 08 (810.42-6627.96)	5122.80 (377.31-9428.21)	2504.34 (338.34-4406.849	275.65 (58.33-1046.64)	645.56 (163.59-1453.69)
	**PND 7**	C57BL6/J	3.41 (1.49-29.21)	4.21 (1.51-11.21)	2.57 (2.15-15.87)	4.45 (1.82-15.50)	2.83 (1.74-12.32)	2.11 (0.55-12.30)
		BTBR	5005039.32 (40516.56-8839493.09)	5643.89 (88.11-53558.12)	2766.45 (244.07-22342.22)	688.14 (262.87-3016.69)	665.11 (117.93-3231.60)	581.59 (24.24-3633.68)
	**PND 23**	C57BL6/J	16.74 (4.05-75.31)	1.32 (0.14-6.57)	2.43 (0.17-7.38)	1.44 (0.16-9.32)	2.01 (0.16-4.60)	2.08 (0.15-5.24)
		BTBR	22839.51 (8465.44-131994.98)	778.40 (68.09-2811.61)	2793.92 (97.04-5438.71)	565.17 (58.63-2304.37)	191.82 (16.14-484.95)	61.50 (20.21-668.48)
	**PND 120**	C57BL6/J	57.43 (11.23-86.05)	0.22 (0.13-0.42)	19.33 (16.44-41.89)	0.03 (0.03-0.08)	8.49 (5.63-18.55)	19.63 (18.32-26.57)
		BTBR	16247900.80 (5763050.67-20078866.80)	10586.95 (7755.76-23383.41)	90303.86 (30814.41-134161.89)	10217.47 (2510.54-19009.77)	58911.02 (31782.57-100689.42)	30257.39 (7121.51-37646.55)
**Whole embryos**								
	**24 h pt[#]**	Vehicle	1.58 (0.42-2.75)	1.78 (0.30-3.17)	1.46 (0.26-3.73)	3.03 (0.46-4.59)	1.65 (0.42-5.90)	1.54 (0.57-8.10)
	**3h pt**	VPA	6.35 (2.72-25.62)	2.86 (1.47-8.54)	2.92 (0.49-13.76)	35.38 (16.34-143.44)	46.25 (11.99-105.35)	56.19 (12.64-146.21)
	**24 h pt**	VPA	7.29 (1.69-29.30)	2.75 (1.40-11.31)	3.33 (1.22-14.88)	40.56 (1.84-78.65)	59.94 (3.54-128.35)	55.88 (3.44-168.13)
**Blood**								
	**PND 1**	Vehicle	2.06 (0.27-4.80)	0.76 (0.33-4.26)	1.56 (0.50-7.30)	0.92 (0.34-4.08)	0.68 (0.32-2.83)	1.62 (0.52-3.58)
		VPA	192.60 (118.68-245.42)	48.83 (25.56-78.61)	71.09 (15.63-114.83)	6.30 (3.28-11.34)	119.00 (49.60-196.22)	252.58 (126.53-346.22)
	**PND 7**	Vehicle	14.93 (5.78-44.60)	0.65 (0.25-1.21)	0.05 (0.01-0.14)	0.15 (0.04-0.60)	2.86 (1.30-5.54)	24.72 (11.01-62.93)
		VPA	32.00 (10.84-65.38)	3.60 (0.86-12.66)	8.20 (2.89-13.58)	0.80 (0.18-2.26)	24.34 (6.77-130.99)	43.27 (22.25-79.83)
	**PND 23**	Vehicle	7.06 (6.58-12.71)	4.74 (1.93-8.20)	1.96 (1.13-6.17)	3.78 (1.55-5.68)	15.19 (10.70-30.02)	143.68 (42.91-255.86)
		VPA	23.78 (14.64-44.60)	6.70 (3.92-13.78)	32.03 (18.81-38.16)	0.75 (0.38-1.16)	37.82 (22.34-58.22)	41.69 (26.18-106.64)
	**PND 120**	Vehicle	0.22 (0.04-4.37)	1.74 (0.46-9.24)	0.02 (0.01-0.09)	0.40 (0.14-1.00)	48.54 (30.37-306.59)	23.25 (9.90-44.74)
		VPA	0.74 (0.11-4.70)	5.84 (1.66-18.50)	0.09 (0.06-0.15)	0.85 (0.58-19.12)	90.66 (26.88-284.00)	36.32 (18.15-149.50)
**Brain**								
	**PND 1**	Vehicle	0.72 (0.56-1.78)	1.22 (0.10-11.15)	1.38 (0.29-2.60)	4.11 (0.03-27.57)	0.89 (0.09-7.68)	0.65 (0.40-3.85)
		VPA	360.38 (91.14-654.77)	938.78 (320.38-1887.11)	151.03 (27.55-404.21)	874.60 (185.27-2461.36)	199.11 (58.62-333.44)	192.82 (51.64-663.14)
	**PND 7**	Vehicle	0.84 (0.38-1.79)	0.46 (0.21-4.27)	1.34 (0.62-5.13)	0.20 (0.03-13.80)	0.71 (0.25-2.02)	0.63 (0.21-2.21)
		VPA	137.11 (38.72-230.00)	390.00 (44.63-899.42)	80.45 (50.19-168.57)	101.93 (2.58-1521.95)	217.44 (19.82-325.03)	211.92 (31.66-561.95)
	**PND 23**	Vehicle	0.47 (0.20-2.00)	0.50 (0.16-6.88)	0.92 (0.15-2.53)	1.31 (0.03-13.47)	0.40 (0.10-3.01)	0.75 (0.22-5.51)
		VPA	51.43 (5.51-90.25)	99.20 (38.83-580.73)	94.78 (58.12-810.97)	29.60 (13.61-128.13)	30.26 (4.90-115.00)	33.90 (3.40-60.62)
	**PND 120**	Vehicle	2.08 (0.99-5.11)	4.61 (0.14-10.97)	0.92 (0.64-1.61)	11.71 (0.05-23.71)	4.09 (0.50-12.67)	3.50 (0.40-8.82)
		VPA	358.65 (76.80-714.76)	830.81 (558.00-1428.24)	250.41 (133.14-385.06)	578.75 (186.27-2578.15)	295.46 (220.32-423.74)	388.58 (183.29-718.26)

[#] hours post treatment
*** median value (IQR)**

Supplementary table S2. Median value and interquartile range (IQR) of the cytokines and TLRs expression levels in C57BL6/J, BTBR, vehicle- and VPA-treated mice

			IL-1β	IL-6	TNF-α	TLR3	TLR4
**Whole embryos**							
	**GD 10.5**	C57BL6/J	1.00 (0.61-1.96)*	1.28 (0.64-1.86)	1.50 (0.23-4.80)	1.26 (0.24-4.17)	0.88 (0.43-2.95)
	**GD 10.5**	BTBR	23.32 (16.19-29.80)	10.82 (6.77-15.41)	62.12 (54.67-103.43)	10.24 (7.83-15.75)	9.45 (5.86-16.72)
**Blood**							
	**PND 1**	C57BL6/J	3.57 (0.28-8.36)	1.48 (1.15-1.86)	3.21 (0.08-8.14)	1.13 (0.78-2.94)	1.89 (0.72-2.41)
		BTBR	269.63 (66.62-362.14)	584.05 (96.65-984.63)	63.48 (19.49-329.68)	573.66 (289.40-44726.06)	177.46 (78.33-1239.22)
	**PND 7**	C57BL6/J	5.10 (1.55-6.47)	0.43 (0.28-4.18)	0.71 (0.24-33.33)	33.24 (13.22-44.46)	8.55 (3.18-33.32)
		BTBR	706.57 (75.20-1794.54)	13.02 (5.30-456.88)	291.29 (43.51-698.40)	208.47 (144.34-1441.79)	137.94 (102.46-1093.88)
**Brain**							
	**PND 1**	C57BL6/J	1.80 E-03 (1.40 E-03-2.50 E-03)	0.18 (0.16-0.20)	0.07 (0.04-0.10)	2.38 (2.20-4.24)	0.23 (0.16-0.28)
		BTBR	182.24 (109.97-258.97)	10.72 (7.88-24.46)	26.44 (20.22-45.54)	871.91 (313.69-1777.77)	68.81 (34.11-333.76)
	**PND 7**	C57BL6/J	1.11 E-03 (5.98 E-06-1.68 E-03)	0.04 (0.01-0.14)	0.05 (0.03-0.07)	3.30 (1.99-11.32)	0.17 (0.08-0.99)
		BTBR	73.41 (26.26-159.26)	90.58 (44.82-139.85)	156.94 (110.96-211.43)	3240.09 (1679.00-3983.41)	101.33 (99.60-201.27)
**Whole embryos**							
	**24 h pt#**	Vehicle	1.00 (0.17-15.10)	2.15 (0.26-23.86)	1.97 (0.10-5.05)	0.95 (0.87-1.22)	0.99 (0.78-1.45)
	**3h pt**	VPA	24.37 (9.43-40.00)	4.05 (0.16-20.86)	7.49 (2.66-14.28)	0.69 (0.52-1.72)	0.84 (0.10-1.98)
	**24 h pt**	VPA	44.10 (23.43-81.43)	87.31 (59.80-104.86)	20.62 (8.99-43.89)	1.33 (0.65-1.72)	2.38 (1.68-2.83)
**Blood**							
	**PND 1**	Vehicle	1.34 (0.16-2.95)	1.43 (0.67-2.31)	2.89 (0.37-5.46)	1.59 (0.30-3.04)	1.35 (0.29-2.74)
		VPA	18787.27 (1632.82-49412.83)	25.55 (11.50-76.98)	42.59 (15.39-148.26)	1.39 (0.51-5.14)	2.73 (2.02-31.45)
	**PND 7**	Vehicle	128.53 (4.38-411.96)	1.83 (0.06-2.40)	0.66 (0.03-0.97)	0.31 (0.05-0.58)	0.63 (0.06-2.09)
		VPA	26087.30 (684.65-115913.87)	46.64 (6.54-135.20)	179.21 (13.53-539.35)	6.82 (0.12-16.31)	13.28 (0.41-26.26)
**Brain**							
	**PND 1**	Vehicle	1290.96 (115.42-2902.06)	0.27 (0.02-0.68)	1.54 (0.70-2.01)	4.11 (2.520-5.53)	102.61 (55.02-166.70)
		VPA	842860.55 (549485.68-1554235.28)	76.06 (30.04-189.13)	379.61 (242.99-823.86)	631.09 (437.87-2408.08)	840.79 (284.24-1551.41)
	**PND 7**	Vehicle	304.32 (50.49-3552.76)	0.46 (0.13-0.90)	1.92 (0.55-3.51)	4.32 (3.25-76.03)	45.06 (35.19-155.17)
		VPA	47323.96 (26011.95-366455.98)	148.80 (4.28-281.63)	343.57 (53.11-553.99)	947.39 (157.21-2857.32)	4178.18 (869.87-11594.55)

# hours post treatment

*** median value (IQR)**

Supplementary table S3. *p*-values obtained by Mann Witney *U* test for the comparison of ERVs', cytokines' and TLRs' transcriptional levels in C57BL6/J, BTBR, vehicle- and VPA-treated mice

		ETnI	ETnII-α	ETnII-β	ETnII-γ	MusD	IAP	IL-1β	IL-6	TNF-α	TLR3	TLR4
**C57BL6/J *vs* BTBR**												
**Whole embryos**	GD 10.5	0.002*	0.002	0.002	0.002	0.002	0.002	0.002	0.002	0.002	0.003	0.003
**Blood**	PND 1	0.019	0.002	0.002	0.012	0.001	0.001	0.008	0.008	0.008	0.008	0.008
	PND 7	0.019	0.038	0.029	0.267	0.000	0.000	0.008	0.016	0.032	0.008	0.008
	PND 23	0.000	0.000	0.000	0.003	0.007	0.000	x	x	x	x	x
	PND 120	0.008	0.008	0.008	0.008	0.008	0.008	x	x	x	x	x
**Brain**	PND 1	0.003	0.003	0.003	0.003	0.003	0.003	0.016	0.016	0.028	0.016	0.016
	PND 7	0.000	0.000	0.028	0.008	0.001	0.002	0.008	0.008	0.008	0.008	0.008
	PND 23	0.000	0.000	0.000	0.000	0.000	0.000	x	x	x	x	x
	PND 120	0.008	0.008	0.008	0.008	0.008	0.008	x	x	x	x	x
**C57BL6/J**												
**Blood**	PND 1 *vs* PND 7	0.421	0.056	0.548	0.032	0.310	0.222	0.841	0.151	0.690	0.008	0.095
	PND 1 *vs* PND 23	0.017	0.126	0.537	0.247	0.004	0.052	x	x	x	x	x
	PND 1 *vs* PND 120	0.008	0.008	0.008	0.008	0.008	0.008	x	x	x	x	x
	PND 7 *vs* PND 23	1.000	0.126	0.329	0.126	0.247	0.792	x	x	x	x	x
	PND 7 *vs* PND 120	0.008	0.008	0.008	0.008	0.008	0.008	x	x	x	x	x
	PND 23 *vs* PND 120	0.004	0.004	0.004	0.004	0.004	0.004	x	x	x	x	x
**Brain**	PND 1 *vs* PND 7	0.286	0.050	0.032	0.032	0.063	0.730	0.111	0.111	0.730	0.413	0.905
	PND 1 *vs* PND 23	0.067	1.000	1.000	1.000	1.000	1.000	x	x	x	x	x
	PND 1 *vs* PND 120	0.028	0.016	0.016	0.016	0.016	0.016	x	x	x	x	x
	PND 7 *vs* PND 23	0.792	0.429	0.662	0.429	0.662	0.662	x	x	x	x	x
	PND 7 *vs* PND 120	0.222	0.008	0.095	0.008	0.095	0.095	x	x	x	x	x
	PND 23 *vs* PND 120	0.429	0.537	0.004	0.004	0.017	0.004	x	x	x	x	x
**BTBR**												
**Blood**	PND 1 *vs* PND 7	0.552	0.603	0.295	0.046	0.603	0.000	0.421	0.222	0.421	0.310	0.841
	PND 1 *vs* PND 23	0.035	0.356	0.780	0.905	0.497	0.079	x	x	x	x	x
	PND 1 *vs* PND 120	0.001	0.001	0.001	0.050	0.019	0.001	x	x	x	x	x
	PND 7 *vs* PND 23	0.114	0.085	0.349	0.003	0.020	0.008	x	x	x	x	x
	PND 7 *vs* PND 120	0.000	0.000	0.000	0.000	0.000	0.027	x	x	x	x	x
	PND 23 *vs* PND 120	0.001	0.001	0.001	0.001	0.001	0.001	x	x	x	x	x
**Brain**	PND 1 *vs* PND 7	0.131	0.603	1.000	0.503	0.261	0.824	0.095	0.151	0.008	0.056	0.548
	PND 1 *vs* PND 23	0.182	0.053	0.315	0.079	0.400	0.028	x	x	x	x	x
	PND 1 *vs* PND 120	0.001	0.004	0.001	0.083	0.001	0.001	x	x	x	x	x
	PND 7 *vs* PND 23	0.008	0.132	0.863	0.605	0.099	0.197	x	x	x	x	x
	PND 7 *vs* PND 120	0.090	0.743	0.028	0.019	0.000	0.001	x	x	x	x	x
	PND 23 *vs* PND 120	0.001	0.001	0.013	0.013	0.001	0.001	x	x	x	x	x
**Vehicle *vs* VPA**												
**Whole embryos**	Veh 24h pt *vs* VPA 3h pt[#]	0.000	0.030	0.176	0.000	0.000	0.000	0.005	0.543	0.063	0.342	0.569
	Veh 24h pt *vs* VPA 24h pt	0.002	0.077	0.042	0.002	0.001	0.001	0.002	0.000	0.042	0.780	0.001
	VPA 3h pt *vs* VPA 24h pt	0.959	0.979	0.407	0.437	0.569	0.365	0.057	0.000	0.231	0.549	0.003
**Blood**	PND 1	0.000	0.000	0.000	0.002	0.000	0.000	0.000	0.000	0.000	0.818	0.041
	PND 7	0.401	0.006	0.000	0.006	0.000	0.006	0.000	0.000	0.000	0.180	0.032
	PND 23	0.001	0.198	0.000	0.003	0.028	0.078	x	x	x	x	x
	PND 120	0.225	0.168	0.019	0.003	0.728	0.068	x	x	x	x	x
**Brain**												
	PND 1	0.000	0.000	0.000	0.000	0.000	0.000	0.000	0.000	0.000	0.002	0.004
	PND 7	0.000	0.000	0.000	0.001	0.000	0.000	0.000	0.000	0.000	0.005	0.001
	PND 23	0.000	0.000	0.000	0.010	0.000	0.000	x	x	x	x	x
	PND 120	0.000	0.000	0.000	0.000	0.000	0.000	x	x	x	x	x
**Vehicle**												
**Blood**	PND 1 *vs* PND 7	0.000	0.331	0.000	0.009	0.047	0.000	0.000	0.820	0.392	0.065	0.589
	PND 1 *vs* PND 23	0.000	0.011	0.406	0.110	0.000	0.000	x	x	x	x	x
	PND 1 *vs* PND 120	0.186	0.406	0.000	0.106	0.000	0.001	x	x	x	x	x
	PND 7 *vs* PND 23	0.169	0.000	0.000	0.000	0.000	0.009	x	x	x	x	x
	PND 7 *vs* PND 120	0.000	0.104	0.651	0.243	0.000	0.651	x	x	x	x	x
	PND 23 *vs* PND 120	0.000	0.266	0.000	0.000	0.008	0.003	x	x	x	x	x
**Brain**	PND 1 *vs* PND 7	0.905	1.000	0.720	0.720	0.616	0.550	0.470	0.270	0.376	0.366	0.445
	PND 1 *vs* PND 23	0.270	0.936	0.437	0.810	0.574	0.769	x	x	x	x	x
	PND 1 *vs* PND 120	0.035	0.728	0.728	0.574	0.247	0.247	x	x	x	x	x
	PND 7 *vs* PND 23	0.560	0.980	0.297	0.820	0.527	0.781	x	x	x	x	x
	PND 7 *vs* PND 120	0.013	0.595	0.462	0.374	0.067	0.067	x	x	x	x	x
	PND 23 *vs* PND 120	0.024	0.551	0.799	0.478	0.020	0.160	x	x	x	x	x
**VPA**												
**Blood**	PND 1 *vs* PND 7	0.000	0.000	0.001	0.000	0.050	0.000	0.809	0.973	0.654	0.699	0.937
	PND 1 *vs* PND 23	0.000	0.000	0.266	0.000	0.012	0.000	x	x	x	x	x
	PND 1 *vs* PND 120	0.000	0.001	0.000	0.087	0.979	0.002	x	x	x	x	x
	PND 7 *vs* PND 23	0.786	0.347	0.004	0.976	0.456	0.928	x	x	x	x	x
	PND 7 *vs* PND 120	0.001	0.531	0.000	0.134	0.148	0.820	x	x	x	x	x
	PND 23 *vs* PND 120	0.001	0.936	0.000	0.110	0.087	0.769	x	x	x	x	x
**Brain**	PND 1 *vs* PND 7	0.095	0.036	0.742	0.231	0.595	0.742	0.001	0.728	0.225	0.818	0.093
	PND 1 *vs* PND 23	0.001	0.011	0.81	0.002	0.022	0.004	x	x	x	x	x
	PND 1 *vs* PND 120	0.887	0.843	0.242	0.843	0.178	0.319	x	x	x	x	x
	PND 7 *vs* PND 23	0.033	0.375	0.488	0.458	0.105	0.019	x	x	x	x	x
	PND 7 *vs* PND 120	0.106	0.031	0.013	0.231	0.118	0.231	x	x	x	x	x
	PND 23 *vs* PND 120	0.002	0.016	0.152	0.003	0.001	0.000	x	x	x	x	x

[#] **hours post treatment**

*** *p*-value**

Supplementary table S4. Spearman correlation analysis in C57BL6/J and BTBR mice

			IL-1β		IL-6		TNF-α		TLR3		TLR4	
			Rho	*p*-value	Rho	*p*-value	Rho	*p*-value	Rho	*p*-value	Rho	*p*-value
**Whole embryos GD 10.5**	**C57BL6/J**	**ETnI**	0.300	0.624	0.600	0.285	-0.100	0.873	0.000	1.000	-0.400	0.507
		**ETnII-α**	0.200	0.747	-0.100	0.873	0.500	0.391	-0.100	0.873	-0.400	0.505
		**ETnII-β**	0.200	0.747	-0.100	0.873	0.100	0.873	0.500	0.391	-0.100	0.873
		**ETnII-γ**	0.200	0.747	-0.100	0.873	0.100	0.873	0.500	0.391	-0.100	0.873
		**MusD**	0.200	0.747	-0.100	0.873	0.500	0.391	-0.100	0.873	-0.400	0.505
		**IAP**	0.500	0.391	-0.200	0.747	0.300	0.624	0.200	0.747	-0.200	0.747
	**BTBR**	**ETnI**	0.262	0.531	0.333	0.420	0.238	0.570	0.714*	0.047	0.690	0.058
		**ETnII-α**	0.214	0.610	0.119	0.779	0.214	0.610	-0.262	0.531	-0.310	0.456
		**ETnII-β**	0.717*	0.047	0.405	0.320	0.857**	0.007	0.554	0.170	0.476	0.233
		**ETnII-γ**	0.595	0.120	0.548	0.160	0.714*	0.047	0.571	0.139	0.190	0.651
		**MusD**	0.833*	0.010	0.786*	0.021	0.714*	0.047	0.667	0.071	0.381	0.352
		**IAP**	0.524	0.183	0.286	0.493	0.595	0.120	0.310	0.456	0.071	0.867

* significant correlation

**highly significant correlation

Supplementary table S5. Spearman correlation analysis in vehicle- and VPA-treated CD-1mice

			ETnI		ETnII-α		ETnII-β		ETnII-γ		MusD		IAP	
			Rho	*p*-value	Rho	*p*-value	Rho	*p*-value	Rho	*p*-value	Rho	*p*-value	Rho	*p*-value
**Whole embryos 24h pt[#]**	**Vehicle-treated**	**ETnI**	1.000		0.815**	0.000	0.814**	0.000	0.451*	0.046	0.474*	0.035	0.463*	0.040
		**ETnII-α**	0.815**	0.000	1.000		0.869**	0.000	0.605**	0.005	0.606**	0.005	0.595**	0.006
		**ETnII-β**	0.814**	0.000	0.869**	0.000	1.000		0.654**	0.002	0.737**	0.000	0.714**	0.000
		**ETnII-γ**	0.451*	0.046	0.605**	0.005	0.654**	0.002	1.000		0.899**	0.000	0.874**	0.000
		**MusD**	0.474*	0.035	0.606**	0.005	0.637**	0.000	0.899**	0.000	1.000		0.947**	0.000
		**IAP**	0.463*	0.040	0.595**	0.006	0.714**	0.000	0.874**	0.000	0.947**	0.000	1.000	
**3h pt**	**VPA-treated**	**ETnI**	1.000		0.394*	0.046	0.568**	0.002	-0.399*	0.043	-0.422*	0.032	-0.280	0.166
		**ETnII-α**	0.394*	0.046	1.000		0.586**	0.002	-0.169	0.410	-0.317	0.115	-0.031	0.880
		**ETnII-β**	0.568**	0.002	0.586**	0.002	1.000		-0.309	0.124	-0.376	0.059	-0.311	0.121
		**ETnII-γ**	-0.399	0.043	-0.0169	0.410	-0.309	0.124	1.000		0.875**	0.000	0.707**	0.000
		**MusD**	-0.422*	0.032	-0.317	0.115	-0.376	0.059	0.875**	0.000	1.000		0.703**	0.001
		**IAP**	-0.280	0.166	-0.031	0.880	-0.311	0.121	0.707**	0.000	0.703**	0.000	1.000	
**24h pt**	**VPA-treated**	**ETnI**	1.000		0.744**	0.001	0.756**	0.001	0.465	0.070	0.465	0.070	0.365	0.165
		**ETnII-α**	0.744**	0.001	1.000		0.959**	0.000	0.474	0.064	0.397	0.128	0.324	0.222
		**ETnII-β**	0.756**	0.001	0.959**	0.000	1.000		0.462	0.072	0.391	0.134	0.329	0.213
		**ETnII-γ**	0.465	0.070	0.474	0.064	0.462	0.072	1.000		0.979**	0.000	0.926**	0.000
		**MusD**	0.465	0.070	0.397	0.128	0.391	0.134	0.979**	0.000	1.000		0.941**	0.000
		**IAP**	0.365	0.165	0.324	0.222	0.329	0.213	0.926**	0.000	0.941**	0.000	1.000	

* significant correlation

**highly significant correlation

[#] **hours post treatment**

Supplementary table S6. Spearman correlation analysis in vehicle- and VPA-treated CD-1 mice

			IL-1β		IL-6		TNF-α		TLR3		TLR4	
			Rho	*p*-value	Rho	*p*-value	Rho	*p*-value	Rho	*p*-value	Rho	*p*-value
**Whole embryos** **24h pt#**	**Vehicle-treated**	**ETnI**	-0.552	0.123	-0.536	0.137	-0.603	0.086	0.167	0.668	-0.402	0.284
		**ETnII-α**	-0.310	0.417	0.226	0.559	0.192	0.620	-0.567	0.112	-0.519	0.152
		**ETnII-β**	-0.385	0.306	-0.218	0.574	-0.351	0.354	-0.083	0.831	-0.343	0.366
		**ETnII-γ**	-0.301	0.431	0.209	0.589	0.092	0.814	-0.417	0.265	-0.167	0.667
		**MusD**	0.075	0.847	0.167	0.667	-0.067	0.864	-0.083	0.831	-0.226	0.559
		**IAP**	-0.251	0.515	0.151	0.699	-0.050	0.898	-0.283	0.460	-0.159	0.683
**3h pt**	**VPA-treated**	**ETnI**	0.296	0.377	0.055	0.873	-0.036	0.915	-0.401	0.222	-0.145	0.670
		**ETnII-α**	-0.032	0.926	0.136	0.689	-0.082	0.811	0.374	0.258	0.600	0.051
		**ETnII-β**	-0.046	0.894	0.336	0.312	0.000	1.000	0.506	0.113	0.609*	0.047
		**ETnII-γ**	-0.269	0.424	0.685*	0.029	-0.182	0.593	0.109	0.749	-0.155	0.650
		**MusD**	-0.264	0.432	0.373	0.259	-0.182	0.593	0.150	0.659	-0.118	0.729
		**IAP**	0.191	0.573	-0.018	0.958	0.218	0.519	0.073	0.831	0.136	0.689
**24h pt**	**VPA-treated**	**ETnI**	-0.200	0.580	-0.309	0.385	0.079	0.829	-0.418	0.229	-0.115	0.751
		**ETnII-α**	-0.091	0.803	-0.309	0.385	0.055	0.881	-0.382	0.276	-0.018	0.96
		**ETnII-β**	-0.127	0.726	-0.370	0.293	0.091	0.803	-0.455	0.187	-0.067	0.855
		**ETnII-γ**	0.164	0.651	0.127	0.726	0.248	0.489	-0.576	0.082	-0.139	0.701
		**MusD**	0.176	0.627	0.200	0.580	0.248	0.489	-0.636*	0.048	-0.248	0.489
		**IAP**	0.079	0.829	0.164	0.651	0.164	0.651	-0.479	0.162	-0.030	0.934
**Brain** **PND 1 + PND 7**	**Vehicle-treated**	**ETnI**	0.488*	0.013	0.135	0.519	0.078	0.712	-0.099	0.748	0.017	0.957
		**ETnII-α**	0.552**	0.004	0.292	0.157	0.178	0.395	0.006	0.986	0.039	0.901
		**ETnII-β**	-0.027	0.898	0.158	0.449	0.122	0.563	-0.105	0.734	-0.028	0.929
		**ETnII-γ**	0.546**	0.005	0.138	0.509	0.052	0.804	-0.162	0.596	-0.003	0.993
		**MusD**	0.523**	0.007	0.278	0.179	0.227	0.275	-0.237	0.436	0.121	0.694
		**IAP**	0.461*	0.020	0.028	0.893	0.138	0.509	-0.525	0.065	-0.113	0.714
	**VPA-treated**	**ETnI**	0.223	0.284	-0.256	0.216	0.351	0.086	-0.0135	0.914	-0.224	0.484
		**ETnII-α**	0.455*	0.022	0.318	0.122	-0.002	0.993	0.168	0.602	0.196	0.697
		**ETnII-β**	0.032	0.880	-0.083	0.693	0.083	0.694	-0.154	0.633	0.028	0.931
		**ETnII-γ**	0.466*	0.019	0.059	0.778	-0.090	0.670	-0.469	0.124	-0.224	0.484
		**MusD**	0.715**	0.000	0.063	0.764	-0.087	0.678	-0.552	0.063	-0.699*	0.011
		**IAP**	0.420*	0.037	-0.151	0.472	0.117	0.579	-0.510	0.090	-0.091	0.779

* significant correlation

**highly significant correlation

hours post treatment
